# Clinical relevance and mechanistic investigation of miR-1908-5p as a prognostic biomarker in colorectal cancer

**DOI:** 10.1186/s41065-026-00645-2

**Published:** 2026-01-27

**Authors:** Chenchen Yuan, Shaofei Chen, Qianli Liu, Riwei Wang

**Affiliations:** 1 The Geriatric Department, Affiliated Jinhua Hospital, Zhejiang University School of Medicine, Jinhua, 321000 China; 2 Department of Gastrointestinal Surgery, Union Hospital, Tongji Medical College, Huazhong University of Science and Technology, Wuhan, 430022 China; 3 Jiamusi University, Jiamusi, 154007 China; 4 Three Branches of General Surgery, Jiujiang City Key Laboratory of Cell Therapy, Jiujiang NO.1 People’s Hospital, Jiujiang, 332000 China

**Keywords:** MiR-1908-5p, Colorectal cancer, SCAMP4, Prognostic marker

## Abstract

**Background:**

Colorectal cancer (CRC) is characterized by a complex pathogenesis and substantial heterogeneity in prognosis.

**Objective:**

This study aims to elucidate the clinical significance of miR-1908-5p in CRC, as well as its association with clinicopathological parameters and patient prognosis.

**Method:**

miR-1908-5p expression in tumor tissues was quantified by RT-qPCR. Survival outcomes were assessed using the Kaplan–Meier method. The chi-square (χ^2^) test and Cox regression analysis were employed to evaluate clinicopathological correlations and prognostic value.miR-1908-5p was compared between NCM460 and CRC lines (HT-29, HCT116), overexpressed, and functionally tested by CCK-8 and Transwell assays. TargetScan and luciferase assay verified the targeting of SCAMP4 3'-UTR. qPCR and Pearson analysis defined their correlation in CRC.

**Results:**

miR-1908-5p is downregulated in CRC tumors versus adjacent normal mucosal tissues. Low expression predicts poor prognosis and is associated with reduced survival rates in colorectal cancer, correlating with advanced TNM stage and elevated serum CA19-9 levels. CRC lines (HT-29 and HCT116) show reduced miR-1908-5p compared to NCM460. miR-1908-5p mimic suppresses proliferation, migration, and invasion. TargetScan-predicted binding to SCAMP4 3'-UTR was validated by dual-luciferase reporter assay. miR-1908-5p overexpression lowers SCAMP4, which is overexpressed in tumor tissues and exhibits an inverse correlation with miR-1908-5p.

**Conclusion:**

miR-1908-5p is downregulated in CRC, correlates with TNM stage, nodal metastasis, elevated CA19-9 levels, and poor survival, and suppresses CRC cell proliferation, migration, and invasion by directly targeting SCAMP4, thereby qualifying as a potential prognostic biomarker for CRC.

## Introduction

Colorectal cancer (CRC), a malignant neoplasm consistently ranking among the top three globally in terms of incidence and mortality, has become a major public health challenge threatening human health [1–3]. In recent years, despite significant advances in the diagnosis and management of CRC, patient survival rates remain low and the overall prognosis remains poor, highlighting the urgent need for more effective therapeutic strategies [4, 5]. The primary underlying reason is the complex pathogenesis of CRC and the current lack of effective biomarkers for early diagnosis and prognostic evaluation [6, 7]. Therefore, in-depth investigation of the key molecular regulatory mechanisms involved in CRC initiation and progression, along with the identification of clinically valuable molecular markers and therapeutic targets, represents a critical direction in contemporary CRC research.

A growing body of evidence indicates that aberrant microRNA (miRNA) expression is closely associated with tumor initiation and cancer progression [8]. Within malignant tumors, (miRNAscan act as either tumor suppressors or oncogenes [9]. Several miRNAs have been validated as potential molecular biomarkers for tumor diagnosis, prognosis prediction, and targeted therapy [10]. Previous studies have shown that miR-1908 is aberrantly expressed in multiple malignancies [11]. For instance, in cervical cancer, miR-1908 specifically targets the HDAC10 gene, thereby promoting cancer cell invasiveness [12]. In glioma, miR-1908 correlates with poor prognosis by enhancing cell proliferation, invasion, and anti-apoptotic capacity, as well as regulating the SPRY4/RAF1 signaling axis [13]. In prostate cancer, miR-1908-5p functions as an inhibitory factor by targeting the SRM gene, a process that directly modulates extracellular vesicle secretion [14]. In CRC, miR-1908-5p exhibits a marked reduction in tumor tissues compared to adjacent non-tumor tissues [15], suggesting a potential tumor-suppressive role in this malignancy. However, its precise clinical significance, downstream targets, and underlying molecular mechanisms in CRC remain incompletely understood and thus warrant further investigation.

Based on the aforementioned background, this study aims to elucidate the expression pattern of miR-1908-5p in CRC and its association with clinicopathological characteristics and patient prognosis via analysis of clinical samples. Furthermore, functional effects of miR-1908-5p on CRC cells will be investigated using cell-based and molecular biology assays, with the objective of providing a novel theoretical basis and experimental foundation for improved prognosis assessment and targeted therapy in CRC.

## Patients and methods

### Research subjects and clinical sample collection

A total of 110 CRC patients who underwent radical surgical resection at Jiujiang City Key Laboratory of Cell Therapy, Jiujiang No.1 People’s Hospital between January 2015 and December 2018 were enrolled in this study. All diagnoses were confirmed by postoperative histopathological examination, and none of the included patients had received preoperative radiotherapy, chemotherapy, or targeted therapy. Patients with a history of other malignancies were excluded from the study. During surgery, tumor tissues and adjacent non-tumor tissues (sampled at least 5 cm from the tumor margin, with histological confirmation of no tumor cell infiltration) were collected.

Comprehensive clinical data were systematically documented, including gender, age, tumor location, postoperative pathological characteristics (TNM stage, lymph node metastasis), serum CA19-9 levels, and follow-up outcomes (vital status and survival time).

### Cell culture and transfection

The normal human colonic epithelial cell line NCM460, CRC cell lines HT-29 and HCT116, and 293T cells were obtained from the Cell Bank of the Chinese Academy of Sciences. NCM460, HT-29, and HCT116 cells were cultured in DMEM medium (12,491,015, Thermo Fisher Scientific) supplemented with 10% fetal bovine serum (FBS, A5670701, Thermo Fisher Scientific), 100 U/mL penicillin–streptomycin (15,140,148, Thermo Fisher Scientific), whereas 293 T cells were maintained in RPMI-1640 medium (11,875,093, Thermo Fisher Scientific) containing 10% FBS.

Cell transfection was performed when cells reached 70–80% confluence, using Lipofectamine 3000 (L3000015, Thermo Fisher Scientific) following the manufacturer's protocol.

### Detection of miR-1908-5p and SCAMP4 mRNA expression levels

Total miRNA was isolated from tissues and cells using a commercial miRNA extraction kit (DP501, TIANFEN), and total RNA was extracted using a total RNA isolation kit(R0018S, Beyotime). cDNA was synthesized via reverse transcription according to the manufacturer’s instructions for the RT-qPCR kit (RR037A, TaKaRa). U6 small nuclear RNA (snRNA) was used as the internal reference gene for miR-1908-5p quantification, and glyceraldehyde-3-phosphate dehydrogenase (GAPDH) was used as the internal reference gene for SCAMP4 mRNA normalization.

### Clinical prognostic analysis

Based on the median expression level of miR-1908-5p in CRC tissues, the 110 enrolled patients were divided into a low-expression group (n = 57) and a high-expression group (*n* = 53). Overall survival (OS) was estimated using the Kaplan–Meier method.

### CCK-8 assay

After transfection, HT-29 and HCT116 cells were seeded in 96-well plates at a density of 5 × 10^3^ cells per well. At 0, 24, 48, and 72 h after seeding, 10 μL of CCK-8 solution (40203ES60, YEASEN) was added to each well. Following an additional 2-hour incubation at 37°C with 5% CO₂, the absorbance (OD value) at 450 nm was measured. This experiment was independently repeated three times, with three technical replicates established in each run.

### Transwell assays for cell migration and invasion

Migration Assay: Twenty-four hours post-transfection, HT-29 and HCT116 cells were resuspended in serum-free medium to a final concentration of 1 × 10^5^ cells/mL. A 200 μL aliquot of the cell suspension was added to the upper chamber of a Transwell insert, while the lower chamber was filled with 600 μL of medium containing 20% FBS. Following incubation at 37 °C with 5% CO₂ for 24 h, non-migrated cells adhering to the upper surface of the membrane were gently removed using a cotton swab. Cells that had migrated to the lower surface of the membrane were fixed with 4% paraformaldehyde for 30 min, followed by staining with 0.1% crystal violet for 15 min.

Invasion Assay: Matrigel matrix gel was pre-diluted to a 1:8 ratio with serum-free medium. Fifty microliters of the diluted Matrigel was evenly coated onto the upper chamber of the Transwell insert and allowed to solidify at 37 °C for 30 min. Subsequent experimental procedures were identical to those described for the migration assay.

The aforementioned migration and invasion experiments were independently repeated three times, with three technical replicates established in each run.

### Dual-luciferase reporter assay

miR-1908-5p mimics, miR-1908-5p inhibitors, and their respective negative controls were co-transfected with either SCAMP4-WT (wild-type) or SCAMP4-MUT (mutant) plasmids. The relative luciferase activity was measured using a dual-luciferase reporter assay kit(JKR23008, GeneCreate).

### Statistical analysis

All statistical analyses and graphical presentations were performed using GraphPad Prism software. Quantitative data are expressed as the mean ± standard deviation (x̄ ± SD). For comparisons between two groups, the independent-samples t-test or paired t-test was used as appropriate. For multiple group comparisons in single-factor designs, one-way analysis of variance (ANOVA) was employed, followed by post hoc tests (e.g., Tukey’s test) if significant differences were detected. Two-way ANOVA was used to analyze multi-factor experiments. Categorical variables are presented as frequencies, and intergroup comparisons were performed using the chi-square (χ^2^) test. Linear correlations between continuous variables were evaluated using Pearson correlation analysis. A *P* value < 0.05 was considered statistically significant.

## Results

### Levels of miR-1908-5p expression in CRC

**Fig. 1:**
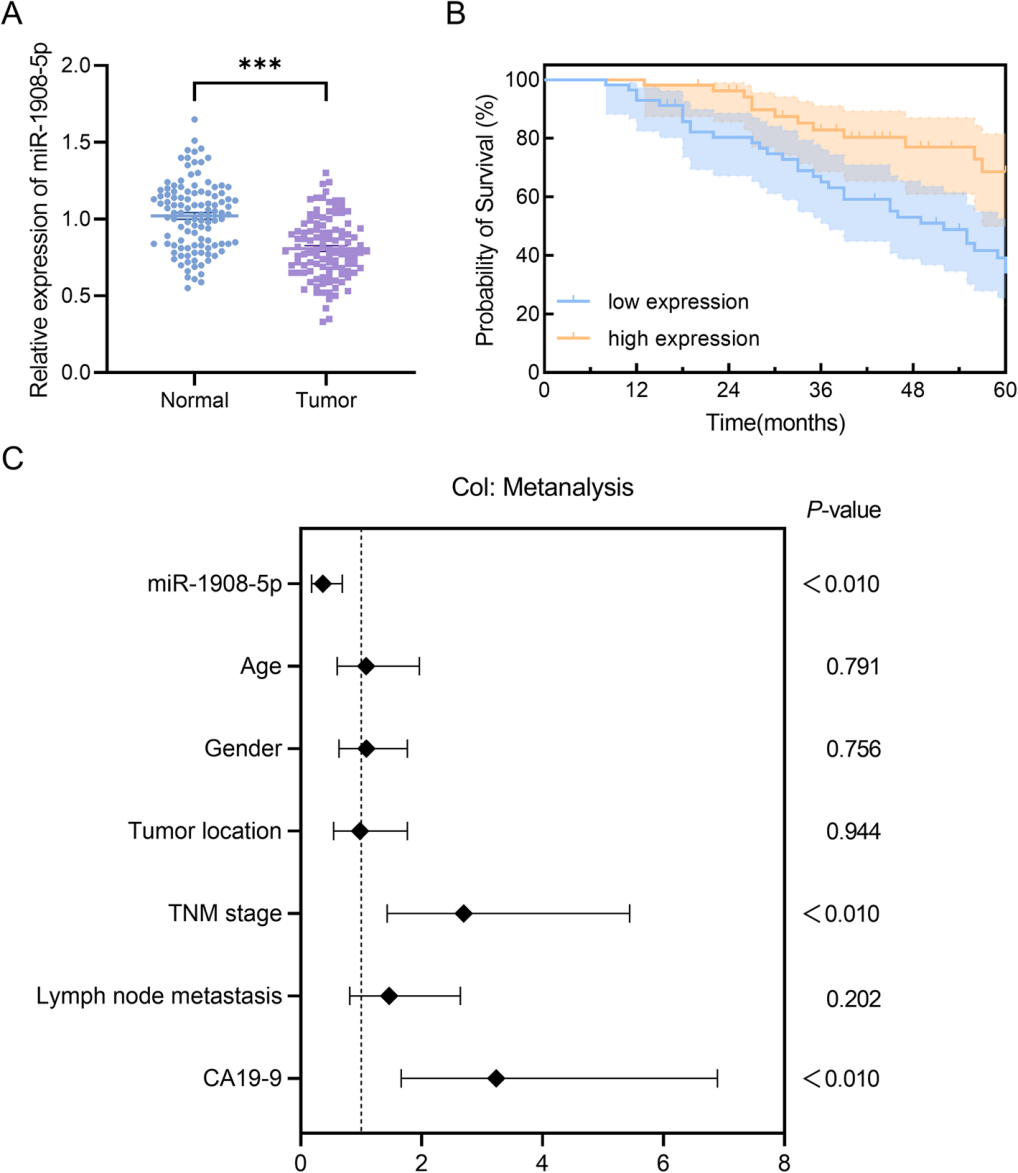
Expression of miR-1908-5p in CRC tissues and its association with patient prognosis. **A** miR-1908-5p expression is significantly lower in CRC tumor tissues compared to normal tissues, ***P < 0.001. **B** Kaplan–Meier survival analysis reveals that patients with low miR-1908-5p expression have significantly worse OS, *P* < 0.01. **C** Multivariate Cox regression analysis identifies low miR-1908-5p expression (HR = 0.365, 95% CI: 0.181–0.688, *P* < 0.01), TNM stage (HR = 2.695, 95% CI: 1.431–5.439, *P* < 0.01), and CA19-9 ≥ 37 U/mL (HR = 3.232, 95% CI: 1.662–6.893, *P* < 0.01) as independent risk factors for poor prognosis in CRC patients

The relative expression level of miR-1908-5p was significantly lower in CRC tumor tissues than in adjacent non-tumor tissues (*P* < 0.01, Fig. 1A).

### Association between miR-1908-5p levels and clinical pathological features

The chi-square test demonstrated that miR-1908-5p expression was significantly correlated with TNM stage, lymph node metastasis, and CA19-9 levels in CRC patients (*P* < 0.001), whereas no statistically significant associations were observed with age, gender, or tumor location (*P* > 0.05, Table 1).

**Table 1:** Association of miR-1908-5p expression with clinicopathologic characteristics of colorectal cancer

Parameters	Low-expression group(n = 57)	High-expression group(n = 53)	χ^2^	*P*-value
Age (years)			1.784	0.180
≤ 60	32	23		
> 60	25	30		
Gender			0.642	0.420
Male	29	31		
Female	28	22		
Tumor location			0.572	0.450
Colon	26	28		
Rectum	31	25		
TNM stage			14.450	< 0.001
Ⅰ-Ⅱ	17	35		
Ⅲ	40	18		
Lymph node metastasis			25.930	< 0.001
No	20	44		
Yes	37	9		
CA19-9(U/ml)			28.410	< 0.001
< 37	12	38		
≥ 37	45	15		

### miR-1908-5p expression and prognosis in CRC patients

Kaplan–Meier survival analysis demonstrated that CRC patients in the miR-1908-5p low-expression group had significantly shorter OS than those in the high-expression group (*P* < 0.01, Fig. 1B).

Furthermore, univariate and multivariate Cox regression analyses were performed to identify independent prognostic factors for CRC patients. The results revealed that miR-1908-5p expression level, TNM stage, and CA19-9 level were all independently associated with the prognosis of CRC patients (*P* < 0.01, Fig. 1C).

### miR-1908-5p expression in CRC cell lines and transfection efficiency validation

**Fig. 2:**
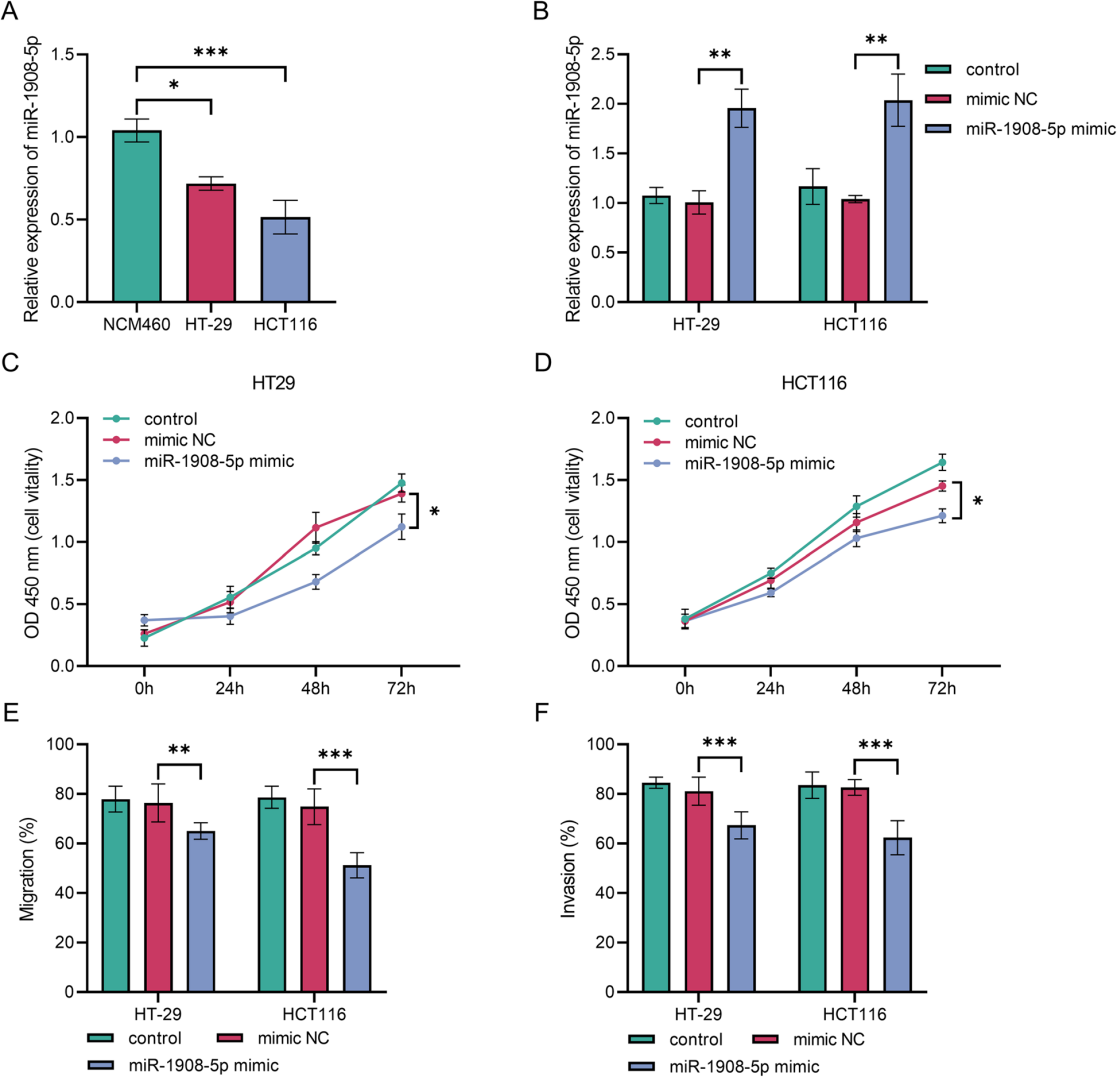
Expression of miR-1908-5p in CRC cell lines and its functional effects on colorectal cancer cells. **A** miR-1908-5p expression is significantly downregulated in CRC cell lines HT-29 and HCT116 compared to normal control cells, ***P < 0.001. **B** Transfection with miR-1908-5p mimic effectively increases miR-1908-5p expression levels in both cell lines, **P < 0.01. **C** Overexpression of miR-1908-5p significantly suppressed the proliferation of HT-29 cells, *P < 0.05. **D** Overexpression of miR-1908-5p significantly suppressed the proliferation of HCT116 cells, *P < 0.05. E: miR-1908-5p overexpression markedly inhibits the migration capacity of both HT-29 and HCT116 cells, ***P < 0.001. F: miR-1908-5p overexpression markedly inhibits the invasive potential of both HT-29 and HCT116 cells, ***P < 0.001

The expression level of miR-1908-5p was significantly lower in the CRC cell lines HT-29 and HCT116 than in NCM460 cells (*P* < 0.001, Fig. 2A). Transfection with the miR-1908-5p mimic significantly increased the relative expression level of miR-1908-5p in HT-29 and HCT116 cells, compared to the control group and mimic NC group (*P* < 0.001, Fig. 2B).

### Effects of miR-1908-5p overexpression on the biological functions of CRC cells

In HT-29 and HCT116 cells, the miR-1908-5p mimic group exhibited significantly lower OD values at 72 h than the control group and mimic NC group (*P* < 0.001, Fig. 2C, 2D).

The migration capacity of HT-29 and HCT116 cells was notably diminished in the miR-1908-5p mimic group (*P* < 0.001, Fig. 2E).

The Transwell invasion assay showed a marked decrease in the invasive capacity of cells in the miR-1908-5p mimic group (*P* < 0.001, Fig. 2F).

### SCAMP4 is identified as a target gene regulated by miR-1908-5p

**Fig. 3:**
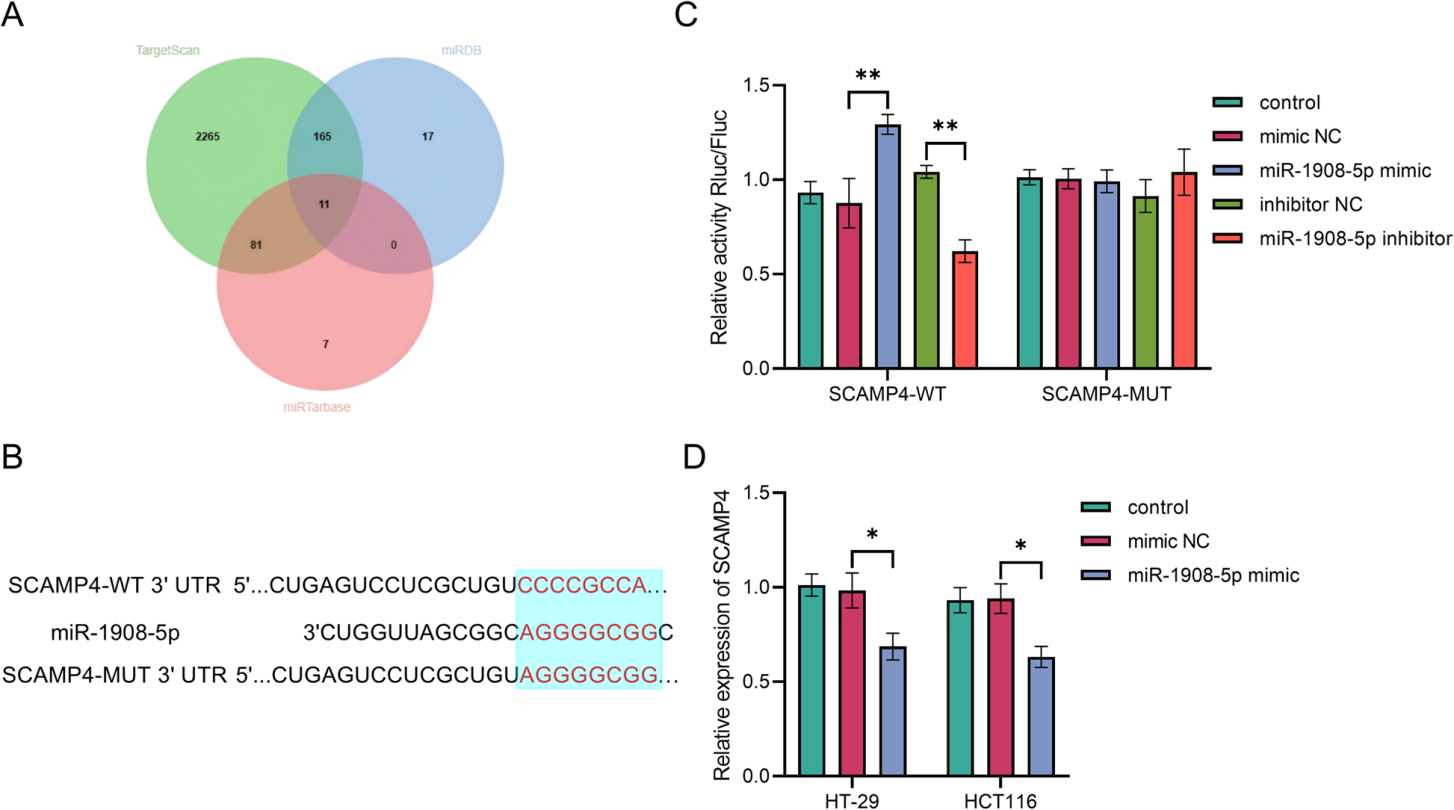
Validation of the direct targeting and binding of miR-1908-5p to SCAMP4. **A** Venn analysis was performed to identify overlapping predicted target genes of miR-1908-5p from the TargetScan, miRDB, and miRTarBase databases. **B** The conserved binding site sequence of miR-1908-5p within the 3'-UTR of SCAMP4, as predicted by the TargetScan database, and the corresponding mutation design in the mutant (MUT) vector. **C** The dual-luciferase reporter assay confirmed that miR-1908-5p directly binds to the 3'-UTR of SCAMP4, **P < 0.01. **D** Overexpression of miR-1908-5p significantly reduced SCAMP4 mRNA expression levels, *P < 0.05, **P < 0.01

To identify the downstream target genes of miR-1908-5p, an integrated bioinformatics analysis was performed using three databases (TargetScan, miRDB, and miRTarBase), yielding 11 overlapping potential target genes (Fig. 3A) Further analysis using TargetScan revealed the conserved binding site between miR-1908-5p and the 3'-UTR of SCAMP4, with a relatively high context score (Fig. 3B). Based on these bioinformatic predictions and its biological relevance, SCAMP4 was selected as the primary candidate target gene for further investigation.

Luciferase activity was significantly increased when SCAMP4-WT was co-transfected with the miR-1908-5p mimic (*P* < 0.01) and markedly decreased when co-transfected with the miR-1908-5p inhibitor (*P* < 0.01). No significant alteration in luciferase activity was observed in the SCAMP4-MUT group, though (*P* > 0.05, Fig. 3C).

When examining HT-29 and HCT116 cells, it was found that the relative mRNA level of SCAMP4 was notably reduced in the miR-1908-5p mimic group compared to the control groups (*P* < 0.05, Fig. 3D).

### miR-1908-5p Is Negatively Correlated with SCAMP4

**Fig. 4:**
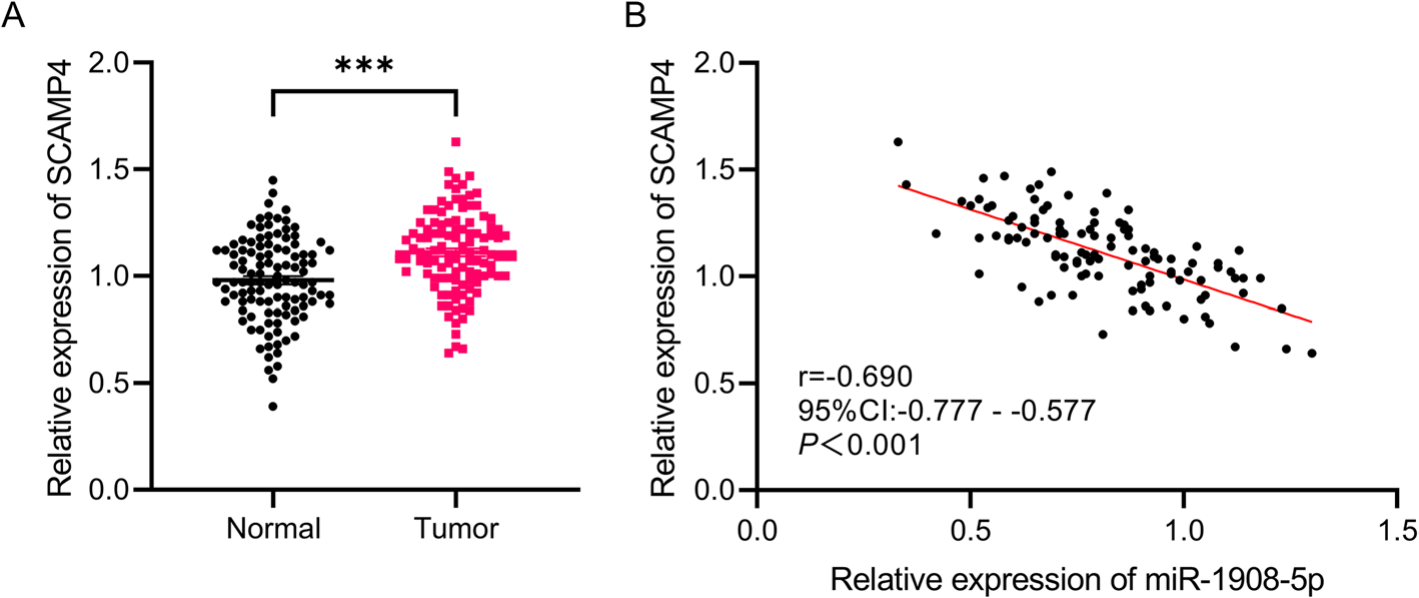
Expression of SCAMP4 in CRC tissues and its correlation with miR-1908-5p. **A** SCAMP4 mRNA expression was significantly upregulated in CRC tumor tissues compared to adjacent normal tissues, ***P < 0.001. **B** Pearson correlation analysis revealed a significant negative correlation between miR-1908-5p and SCAMP4 mRNA expression levels in CRC tumor tissues (*r* = −0.690, 95% CI: −0.777 to −0.577, *P* < 0.001)

Results from RT-qPCR revealed a significant elevation in the relative mRNA level of SCAMP4 in CRC tumor tissues when contrasted with adjacent non-tumor tissues (*P* < 0.01, Fig. 4A).

Results of Pearson correlation analysis showed that miR-1908-5p expression and SCAMP4 mRNA levels exhibited a significant negative correlation in CRC tumor tissues (*P* < 0.001, Fig. 4B).

## Discussion

Accurate prognostic assessment is critical for clinical decision-making in the diagnosis and treatment of CRC [16]. Currently, clinical practice primarily relies on conventional indicators such as TNM stage and histopathological differentiation grade [17, 18]. However, these parameters fail to fully capture the molecular heterogeneity of CRC, potentially leading to inaccurate prognostic predictions in certain patients [19]. For instance, patients with the same TNM stage may exhibit substantial differences in OS, underscoring the limitations of the traditional staging system [20]. In recent years, growing efforts have been devoted to identifying molecular prognostic biomarkers, including DNA mutations (e.g., KRAS, BRAF) and long non-coding RNAs (lncRNAs) such as HOTAIR and MALAT1. However, many of these markers are limited by high detection costs or insufficient specificity, which restricts their widespread application in routine clinical practice [21]. This study demonstrates that miR-1908-5p is significantly downregulated in CRC tissues, and its low expression is strongly associated with advanced TNM stage, lymph node metastasis, and elevated serum CA19-9 levels. CA19-9 is a tumor-associated antigen widely used in the clinical assessment of prognosis in CRC [22]. This suggests that miR-1908-5p has clinical significance comparable to that of CA19-9 and may serve as a potential indicator of disease progression. More importantly, Cox regression analysis confirms that low miR-1908-5p expression is an independent risk factor for adverse prognosis in patients with CRC. This finding is consistent with previous studies on tumor suppressor miRNAs such as miR-21 and miR-92a-3p [23, 24], where reduced expression correlates with poor outcomes in CRC. The present study further establishes the clinical relevance of miR-1908-5p in prognostic stratification, providing a novel molecular marker for improved risk assessment. Furthermore, the RT-qPCR-based detection method is simple, cost-effective, and highly amenable to translation into clinical practice.

MicroRNAs (miRNAs) act as key "molecular switches" in the initiation and progression of CRC, with significant functional duality [25]. Some miRNAs promote tumor development by targeting and downregulating tumor suppressor genes. For example, miR-934 induces M2 macrophage polarization, thereby creating an immunosuppressive tumor microenvironment that facilitates CRC liver metastasis [26]. In contrast, another subset of miRNAs exerts tumor-suppressive effects by inhibiting oncogenes. For instance, miR-144 directly targets and binds to the GSPT1 gene, blocking its positive regulation of the cell cycle and consequently suppressing the proliferation and migration of CRC cells [27]. Recent studies have shown that certain miRNAs can also regulate CRC invasion and metastasis by modulating the tumor microenvironment and epithelial-mesenchymal transition (EMT), making them a focus of research on the molecular mechanisms of CRC [28]. Our results revealed that miR-1908-5p was significantly downregulated in CRC tumor tissues, and its expression levels in the HT-29 and HCT116 CRC cell lines were lower than those in the normal colonic epithelial cell line NCM460. This consistent expression pattern between tissues and cell lines is consistent with the stable regulatory role of miRNAs in tumorigenesis. Functional experiments further confirmed that overexpression of miR-1908-5p markedly inhibits the proliferation, migration, and invasion capacities of CRC cells. This observation is consistent with the tumor-suppressive role of miR-1908 in non-small cell lung cancer (NSCLC) [29], suggesting that its tumor-suppressive function may be conserved across multiple cancer types. These results support the hypothesis that members of the miR-1908 family share a conserved tumor-suppressive mechanism in various solid tumors.

Belonging to the secretory carrier membrane protein (SCAMP) family, SCAMP4 primarily participates in intracellular vesicle transport and signal pathway regulation [30]. In recent years, its roles in various cancers and diseases have attracted increasing attention. Previous studies have demonstrated that SCAMP4 is overexpressed in pancreatic adenocarcinoma (PAAD), where it holds potential as an auxiliary diagnostic marker and prognostic indicator [31]. SCAMP4 is also upregulated in glioma, and its expression level is significantly associated with tumor grade and immune infiltration, with patients exhibiting high expression showing poorer prognosis [32]. This study used dual-luciferase reporter assays to confirm that miR-1908-5p regulates SCAMP4 expression by directly targeting its 3'-UTR, and a notable negative correlation between miR-1908-5p and SCAMP4 was observed in CRC tissues. As a family member, SCAMP4 is consistently overexpressed in glioma, PAAD, and CRC [31, 32]. Based on our functional experimental results, we propose that SCAMP4 mediates vesicle transport in CRC cells, thereby participating in material trafficking and signal transduction, and regulating CRC cell proliferation, migration, and invasion. As a tumor suppressor, miR-1908-5p inhibits the malignant progression of CRC by downregulating SCAMP4 expression and disrupting its pro-tumorigenic signaling pathways. This mechanism not only highlights the oncogenic role of SCAMP4 in CRC but also enhances our understanding of miRNA–mRNA regulatory networks in CRC pathogenesis.

This study has several limitations. First, the sample size is relatively small and the study design is single-center, which may introduce selection bias. Future studies should expand the sample size and conduct multi-center investigations to validate the findings. Second, the expression levels and clinical significance of miR-1908-5p across different molecular subtypes of CRC, such as the consensus molecular subtypes (CMS), were not examined. Given the heterogeneity of CRC subtypes, this may influence the prognostic value and therapeutic relevance of miR-1908-5p. Subsequent analyses should incorporate subtype classification to address this gap. Third, the study primarily relied on in vitro cell experiments and lacked in vivo validation using animal models to confirm the regulatory role of the miR-1908-5p/SCAMP4 axis. Future work should include animal studies to strengthen mechanistic insights. Finally, the specific downstream signaling molecules associated with SCAMP4 were not fully characterized, and the detailed molecular pathways through which SCAMP4 mediates the malignant phenotype of CRC remain to be elucidated. 

In summary, miR-1908-5p is downregulated in CRC tumor tissues and significantly associated with TNM stage, lymph node metastasis, elevated serum CA19-9 levels, and poor prognosis. It suppresses the proliferation, migration, and invasion of CRC cells by directly targeting SCAMP4. The miR-1908-5p/SCAMP4 axis represents a promising prognostic biomarker and a potential therapeutic target in CRC.

## Data Availability

The datasets used and/or analysed during the current study are available from the corresponding author on reasonable request.
